# One size does not fit all: community views on choices for TB treatment and prevention

**DOI:** 10.5588/pha.23.0034

**Published:** 2023-09-21

**Authors:** A. Makone, K. Angami, D. Bhattacharya, M. Frick, J. G. Castillo, R. Herrera, L. McKenna, G. K. Moses, O. Rucsineanu, A. H. Sari, J. Stillo, P. Agbassi

**Affiliations:** 1 Stellenbosch University, Cape Town, South Africa;; 2 Global TB Community Advisory Board, New York, NY, USA;; 3 Access to Rights and Knowledge Foundation, Kohima,; 4 Survivors Against TB, New Delhi, India;; 5 Treatment Action Group, New York, NY;; 6 McGovern Medical School at the University of Texas Health Science Center, Houston, TX, USA;; 7 Moldova National Association of Tuberculosis Patients “SMIT” (Society of Moldova against Tuberculosis), Chis¸ina˘u, Moldova;; 8 Department of Anthropology, Wayne State University, Detroit, MI, USA

**Keywords:** tuberculosis, community views, treatment options, differentiated care delivery

## Abstract

Treatment and prevention paradigms in TB have been dominated by a ‘one-size-fits-all’ approach, in which all persons are given the same treatment regimens. This stands in contrast to other health conditions, where differentiated models of care have been shown to be effective. In this Viewpoint, we make the case for considering multiple factors when deciding which regimens should be offered to people with TB infection and disease. Choice about which regimens to use should be made in conjunction with people who have TB and consider efficacy, safety, duration, pill burden, formulation, drug interactions, time spent in monitoring, drug susceptibility, compatibility with other areas of life, and availability of support services. Ideally, these choices should be considered within an equity framework with the most intensified services being offered to those considered most vulnerable.

## Abstract (fr)

Les paradigmes de traitement et de prévention de la TB ont été dominés par une approche « unique », dans laquelle toutes les personnes reçoivent les mêmes schémas thérapeutiques. Cette approche contraste avec d’autres problèmes de santé, pour lesquels des modèles de soins différenciés se sont avérés efficaces. Dans ce point de vue, nous plaidons en faveur de la prise en compte de multiples facteurs au moment de décider des schémas thérapeutiques à proposer aux personnes atteintes de infection tuberculeuse et de TB maladie. Le choix des traitements doit être fait en collaboration avec les personnes atteintes de TB et tenir compte de l’efficacité, de l’innocuité, de la durée, du nombre de comprimés, de la formulation, des interactions médicamenteuses, du temps consacré à la surveillance, de la sensibilité aux médicaments, de la compatibilité avec d’autres domaines de la vie et de la disponibilité des services d’aide. Idéalement, ces choix devraient être envisagés dans un cadre d’équité, les services les plus intensifs étant proposés aux personnes considérées comme les plus vulnérables.

More than 10 million people become ill with TB each year, but almost half of them are never diagnosed or offered treatment, in part, due to shortcomings of the health systems in which TB services are delivered.^1^ Over the past decade, the TB field has witnessed a blossoming of progress from investments in TB research and drug development.^2^ Although there is still notable ground to cover, there are now multiple options available for the treatment and prevention of TB infection and disease, including shorter, safer, and more tolerable regimens.^3^ The various ongoing and planned TB therapeutic trials likely mean that the future will hold many more treatment and prevention options for impacted communities and the providers and programs that serve them.^4^

While in most circles more choice in TB is cause for optimism, among some policymakers and other stakeholders that are more accustomed to a one-size-fits all approach, there is also concern about having multiple options recommended by the WHO for the treatment and prevention of TB.^5^ The situation is further complicated by the fact that these regimens have not been directly compared and have different trade-offs, making the selection of one over another as the ‘ideal’ regimen in all circumstances unlikely.

Having multiple effective treatment options is the norm for many global health conditions, such as antiretroviral therapy for HIV. There are currently more than 30 antiretroviral medications from eight classes approved for HIV treatment that can be used to construct regimens based on efficacy, safety, treatment history/risk of resistance, as well as a person’s preferences around pill burden, adverse events, tolerability, formulation, and co-administration with other medications. There are also different options available in terms of how medications are delivered, including through adherence clubs, multi-month dispensing models, and other approaches that focus on person-centered support – as a collective approach, this is referred to as differentiated service delivery (DSD). Broadening options based on the latest research is also the norm in HIV prevention, where diversity is encouraged by ongoing research and ‘The Choice Agenda’.^6^

TB, however, has for too long been driven by a ‘one-size-fits-all’ approach. This paradigm strives to keep the treatment and prevention of TB simple, with the rationale being that such an approach will ease program planning, procurement, and service provision, making them less prone to error. It underpins the desire for a ‘universal’ regimen, and, at first glance, is embraced by those working in the TB space as a seemingly responsible approach to the disease. A one-size-fits-all regimen and/or approach, however, stands in conflict with person-centered models of TB care,^7^ an issue of central concern to impacted communities.

Embracing multiple treatment options as part of differentiated models of TB care would involve incorporating various approaches into the management of the disease.^8^ Assuming similar and high efficacy, TB regimens would be selected based on principles of shared decision making between people with TB, TB providers, and TB programs. The different therapeutic options that now exist for TB preventive therapy (TPT) could be viewed as a model. The WHO currently recommends five different TPT regimens: 1) a daily regimen of isoniazid (INH, H) and rifapentine (RPT, P) for 1 month (1HP, based on NCT01404312); 2) a once-weekly regimen of INH and RPT for 3 months (3HP, based on NCT00023452); 3) a daily regimen of INH and rifampicin (RIF, R) available as a pediatric formulation, for 3 months (3HR); 4) a daily regimen of RIF for 4 months (4R, based on NCT 00931736); and 5) a daily regimen of INH for 6–9 months (6H). Currently, TB programs decide which regimens to offer to people in need of TPT, largely based on costs and logistics. In a model based on person-centered care, various options could be discussed with the person needing TPT, considering their preferences as well as the pros and cons of the regimens. For people who value or need rapid completion of TPT and for whom the total time spent on treatment is more important than the daily pill burden, the 1HP regimen could be selected. For people who do not want to take tablets daily, the 3HP regimen might be preferred. For children who are not able to swallow tablets, the 3HR regimen might be ideal. For those who cannot take INH for reasons of toxicity or resistance, the 4R regimen might be selected, and for those who cannot take rifamycins for reasons of resistance, intolerance, or drug–drug interactions, the 6H regimen might be chosen. Programs could then monitor which regimens are being used and why to help with forecasting, procurement, and national planning.

For drug-susceptible TB treatment, there are currently two regimen options recommended by the WHO. One is a daily regimen lasting 6 months, consisting of four drugs (INH, RIF, pyrazinamide [PZA, Z] and ethambutol [EMB, E]) available as a combination tablet (6HREZ) and a pediatric formulation. The other is a 4-month regimen of four drugs not yet available in a fixed-dose combination, and consisting of INH, RPT, moxifloxacin (MFX, M), and PZA (4HPMZ, based on NCT02410772).^9^ Under differentiated service delivery, people with TB should be informed about these options. For persons who prioritize a shorter treatment duration and for whom the daily pill burden is not a pressing concern, the 4HPMZ regimen might be selected, while those who want to take fewer pills daily but are able to take treatment for longer periods of time might want to take the 6HREZ regimen. Those who cannot take MFX because of potential cardiac toxicity or drug–drug interactions might preferentially receive the 6HREZ regimen, while those with vision problems who cannot take EMB would receive the 4HPMZ regimen.

There are now multiple options to be considered as part of differentiated models of care for drug-resistant TB (DR-TB). The WHO currently recommends both a 6-month regimen comprising four drugs (bedaquiline [BDQ], pretomanid [Pa], linezolid [LZD] and MFX, or BPaLM, based on NCT02589782), as well as a 9-month regimen, consisting of seven drugs (BDQ, levofloxacin [LVX]/MFX, clofazimine, ethionamide (or LZD), EMB, PZA, and high-dose INH). Although the BPaLM regimen is likely to be preferred by people with DR-TB,^10^ the all-oral 9-month regimen^11^ could be considered because of its duration, pill burden, and adverse event profile for populations in which Pa has not yet been studied or indicated (i.e., pregnant persons, children), or for people unable to tolerate Pa or LZD. Although not yet reviewed by the WHO, the MDR-END trial conducted in South Korea (NCT02619994) tested a 9-month regimen consisting of four drugs (delamanid, LVX, PZA, and LZD), which could be offered to people with DR-TB with BDQ resistance or intolerance.^12^ The endTB trial, which evaluated 9-month regimens containing BDQ and/or delamanid and up to four companion drugs (LZD, MFX, clofazimine, PZA), will have results later this year (NCT02754765) and may provide options for Pa-free, all-oral shorter regimens. It must be mentioned, however, that regimens containing a daily injection are not acceptable to impacted populations and should not be pursued as viable treatment options in any setting, except in people who need rescue regimens.^13^ Drug susceptibility testing must play an important role in guiding treatment regimens chosen for DR-TB.

Differentiated models of care are important for sub-populations of people with TB, including children, pregnant people, and those with comorbidities. Important considerations for regimen selection in these groups include the availability of child-friendly formulations, or a lack of drug–drug interactions with medications taken as part of concomitant therapy. These groups are often the last to benefit from advances in clinical therapeutics as they do not benefit from equitable and prompt inclusion in TB trials. It is important that their unique preferences and needs for TB treatment regimens are also considered as key parts of person-centered care. Key considerations in the delivery of person-centered care for all people with TB are listed in the Table.

**Table:** Factors to consider in differentiated service delivery for TB (assuming equivalent efficacy)

Factors to consider
Treatment efficacy Treatment safety (risk and type of adverse events) Treatment duration Pill burden Availability of acceptable formulations Time spent engaged in monitoring, follow-up, etc. Interactions with other medications Drug susceptibility profiles Compatibility with other important areas of lifestyle (i.e., work, school, recreation, child care, reproductive health, etc.) Availability of support resources (i.e. counseling, treatment literacy)

When there are multiple options, programs will likely make decisions about regimens based on cost alone, a dangerous precedent which further entrenches inequities in care. Instead, they should make sure all stakeholders are aware of the regimen options and partner with affected populations and individuals to determine their treatment preferences and needs, emphasizing treatment literacy and participatory decision-making. True choice must be part of the future of TB prevention and treatment, not only to take into consideration the individual needs and wishes of people receiving treatment but also to continue to build a robust health system in which TB care can occur. Differentiated service delivery models create flexibility and resiliency, allowing programs to better adapt to health systems’ and communities’ challenges. This was seen during the COVID-19 pandemic, when adapted models of care were needed to address the clinic closures and health systems strains: such adjustments were more seamless where differentiated services were available for HIV.^14^

One-size-fits-all approaches to TB prevention, treatment, and services are short-sighted. They do little to advance equity in service delivery, where people are offered the best available therapy according to their individual risks, needs, and preferences. ‘Equality’ usually means all people have the same treatment, including access to baseline testing, drug selection, and monitoring options. ‘Equity’ means that people with higher risks will have more intensified care, including access to additional monitoring, more effective therapy, and compensatory psychosocial and socioeconomic support so that even with their elevated risk, similar outcomes can be achieved as people with lower risk.

While proponents of universal regimens often tout their ease of delivery and simplified logistics, one only need look at the recent history of the universal approach to TB called DOTS that dominated care in the 1990s to see the folly in such a strategy.^15^ The health systems challenges that currently exist in TB must be addressed with a human rights- and equity-driven approach—as opposed to a logistics or financial one—in which the options are expanded, and people with TB are at the center of decision-making: it is the only way we might actually succeed in “ending” TB.
